# Being Mindful at University: A Pilot Evaluation of the Feasibility of an Online Mindfulness-Based Mental Health Support Program for Students

**DOI:** 10.3389/fpsyg.2020.581086

**Published:** 2021-01-11

**Authors:** Miroslav Světlák, Pavla Linhartová, Terezia Knejzlíková, Jakub Knejzlík, Barbora Kóša, Veronika Horníčková, Kristýna Jarolínová, Klaudia Lučanská, Alena Slezáčková, Rastislav Šumec

**Affiliations:** ^1^Department of Psychiatry, Faculty of Medicine, Masaryk University, Brno, Czechia; ^2^Department of Psychology and Psychosomatics, Faculty of Medicine, Masaryk University, Brno, Czechia; ^3^Department of Psychology, Faculty of Arts, Masaryk University, Brno, Czechia; ^4^First Department of Neurology, Faculty of Medicine, Masaryk University and St. Anne’s University Hospital, Brno, Czechia

**Keywords:** mindfulness, online intervention, self-compassion, emotion regulation, life satisfaction, eHealth

## Abstract

University study can be a life period of heightened psychological distress for many students. The development of new preventive and intervention programs to support well-being in university students is a fundamental challenge for mental health professionals. We designed an 8-week online mindfulness-based program (eMBP) combining a face-to-face approach, text, audio, video components, and support psychotherapy principles with a unique intensive reminder system using the Facebook Messenger and Slack applications in two separate runs (*N* = 692). We assessed the program’s effect on mindful experiencing, perceived stress, emotion regulation strategies, self-compassion, negative affect, and quality of life. The results of the presented pilot study confirmed that eMBP is a feasible and effective tool in university students’ mental health support. The students who completed the eMBP reported a reduction of perceived stress with a large effect size (_*p*_η^2^ = 0.42) as well as a decrease of negative affect experience frequency and intensity (_*p*_η^2^ = 0.31), an increase of being mindful in their life (Five Facet Mindfulness Questionnaire subscales:_*p*_η^2^ = 0.21, 0.27, 0.25, 0.28, 0.28), and a higher rate of self-compassion (_*p*_η^2^ = 0.28) with a medium effect size. A small effect size was found in the frequency of using a cognitive reappraisal strategy (_*p*_η^2^ = 0.073). One new result is the observation of an eMBP effect (_*p*_η^2^ = 0.27) on the decrease in attributed importance to the quality-of-life components replicated in two consecutive runs of the program. The study affirms that mindfulness-based interventions can be effectively delivered in an eHealth form to university students.

## Introduction

Current research and clinical experience show that the time of university study can be a life period of heightened psychological distress for some students (Garlow et al., 2008; Bewick et al., 2010; Keyes et al., 2012). Research suggests that, at any given time, 20–25% of students are stressed (Kumaraswamy, 2013), and 50% of students may experience stress in the form of anxiety and depression (Regehr et al., 2013). Alarming results have been repeatedly documented, especially among medical students (Dahlin et al., 2005; Dyrbye and Shanafelt, 2016). Almost half of them experience severe stress-related symptoms, and 5–10% of them report suicidal ideation during their studies (Dyrbye et al., 2006). A negative relationship between the low mental health of students and their academic performance has been documented (Mortier et al., 2015). The capacity of university advisory services to help students is often limited by the number of sessions. The waiting time for psychotherapy treatment or psychological counseling for non-urgent problems, such as stress associated with studies, is often between 8 and 16 weeks, and some students cannot afford it financially if there is a charge for the service. Long waiting times exceeding 7 months for psychotherapy represent a general problem (Cavanagh, 2014; Beck et al., 2015). Furthermore, face-to-face counseling or psychotherapy is not appropriate for everyone (Wong et al., 2018). Moreover, based on our clinical experience in the outpatient unit of the psychiatry department of a university hospital, many students do not realize that their stress is severe; medical students often believe that severe stress is a normal part of the study, and many students from varied disciplines are ashamed to seek help in a timely manner.

In this context, the development of new approaches to preventive and intervention programs to support well-being in university environments is a fundamental challenge for mental health professionals.

### eHealth Programs to Support the Mental Health of University Students

In the last two decades, information and communication technology have been rapidly incorporated into traditional physical and mental healthcare practices (Andersson, 2018). The term eHealth refers to health services and information delivered or enhanced through the Internet and related technologies (Eysenbach, 2001). This direction of healthcare practice reflects an increasing financial pressure on healthcare budgets across the world to look for effective approaches to delivering healthcare with minimal economic costs and maximum selected population impact (Donker et al., 2015). A recent meta-analysis showed that eHealth programs are also increasingly used by university students, and they are effective and feasible for a range of conditions such as stress, anxiety, depression, sleep problems, well-being, and eating disorders (Harrer et al., 2019; Bolinski et al., 2020). No superiority of specific approaches used in eHealth intervention for students is described. To our knowledge, no meta-analyses comparing active treatments with each other in eHealth intervention have been conducted yet. The low-intensity structured interventions [typically cognitive–behavioral therapy (CBT)] are most suitable for eHealth mental health support (Mulder et al., 2017). For example, Hedman et al. (2012) reported that Internet-delivered CBT produced similar outcomes to conventional face-to-face CBT for various diseases.

While most eHealth programs for students are based on cognitive–behavioral therapy (e.g., Lattie et al., 2019), there is also increasing evidence that eHealth mindfulness-based programs (eMBP) represent a beneficial approach to mental health support for university students (Cavanagh et al., 2013; Kvillemo et al., 2016; Danilewitz et al., 2018; Lee and Jung, 2018).

### Mindfulness in eHealth Context

The effectiveness of face-to-face mindfulness-based interventions on improving mental and physical health has been repeatedly documented in healthy people (Keng et al., 2011; Tomlinson et al., 2018) and in people with various psychiatric and somatic conditions (Goldberg et al., 2017; Rahimi-Ardabili et al., 2018). In terms of eMBP delivery modes, systematic reviews and a recent meta-analysis have documented that eMBPs have a significant impact on depression, anxiety, well-being, mindfulness, and stress reduction (Cavanagh et al., 2014; Fish et al., 2016; Spijkerman et al., 2016).

eMBPs have the potential to deliver the benefits of mindfulness-based programs to previously inaccessible large groups of participants. eMBPs (1) are easily accessible, (2) are anonymous, (3) are available 24/7 to people during the course of their daily life, (4) do not necessarily require the involvement of a therapist educated in mindfulness, (5) are less expensive; and (6) save time (Andersson and Cuijpers, 2009; Andersson and Titov, 2014; Spijkerman et al., 2016). In terms of the university students’ mental health support specifics “mentioned above,” eMBPs seem to be an appropriate tool. The demand for online mindfulness delivery is reflected in the increasing number of mindfulness-based mobile apps (Mani et al., 2015). Almost half of the people in one study would prefer an online format of mindfulness meditation intervention to a face-to-face format (Wahbeh et al., 2014).

eMBPs have been documented as effective in supporting mental health in healthy subjects (Cavanagh et al., 2018; Querstret et al., 2018), in patients with cancer (Zernicke et al., 2014; Kubo et al., 2018), and in patients with depression (Lappalainen et al., 2015), anxiety (Krusche et al., 2013), tinnitus (Hesser et al., 2012), chronic pain (Dowd et al., 2015), and fibromyalgia (Davis and Zautra, 2013).

### Mechanisms of Mindfulness and Beneficial Effects on Well-Being

The new skills obtained in mindfulness-based programs are broad, and they are not associated with any particular syndrome (Hayes et al., 2011). The therapeutic change does not occur through a mechanistic alteration of problematic cognition and behavior (Mulder et al., 2017). It is induced by moving the participants toward a more open, curious, aware, and active approach to dealing with psychological blockages to adaptive living; the result is a broad set of positive life benefits (Hayes et al., 2011). The components proposed to describe the mechanisms through which mindfulness works are attention regulation, body awareness, emotional awareness, emotion regulation, change in perspective on the self, self-compassion, and self-transcendence (Neff, 2003; Hölzel et al., 2011; Vago and David, 2012; Tang et al., 2015; Alsubaie et al., 2017; López et al., 2018). Mindfulness and self-compassion are considered to be transtherapeutic and transdiagnostic phenomena that play roles in the development and maintenance of mental health and quality of life (Schanche, 2013; Greeson et al., 2014). Enhanced emotion regulation, considered a transdiagnostic factor, may underlie many of the beneficial effects of mindfulness meditation (Aldao et al., 2010; Tang et al., 2015). Reappraisal has been suggested to be one of the core emotion regulation strategies during mindfulness practice (Hölzel et al., 2011). Another proposed mechanism of change induced by mindfulness training is value clarification (Shapiro et al., 2006). Mindfulness makes it possible to see clearly what is important for a satisfying life. Through mindfulness training, people start to recognize the infinite and transitory nature of reality and the independence of happiness from external things (Mingyur, 2007).

### Components of Effective and Feasible eMBP

There are important differences in the construction, length, and delivery modes of eMBPs (Fish et al., 2016). These programs vary on the spectrum of delivery modes, ranging from audio CDs combined with regular phone call reminders (Altschuler et al., 2012) to web-based programs combined with periodic email reminders (Krusche et al., 2012; Cavanagh et al., 2018). They also vary between synchronous modes (where the therapist and the client correspond in real time) to asynchronous modes (where the therapist and the client each spend time in some contact during the program, but not necessarily at the same time). Although it cannot yet be concluded which mode of delivery is the most effective because of the enormous heterogeneity of studies, some common factors appear. A review by Fish et al. (2016) revealed some elements that should be included in future programs, such as a modular course structure, use of varied materials within the same course (e.g., text, audio, videos, and printouts), and an e-learning approach. Kelders et al. (2012) also documented the importance of social support by peers through the opportunity to contact others using the same program. Another significant part of eMBP programs is a reminder system. The reminder system through emails, text messages, or messages on a smartphone is a unique option of eHealth technology (Schwebel and Larimer, 2018). For instance, Wells et al. (2020) documented the importance and effectivity of smart messaging in reminding oncology patients in a mindfulness-based cognitive therapy (MBCT) program of prescribed between-session activities. The program completion was eight times greater for patients using smart messaging than for non-users. Appointments, homework reminders, assessment, and feedback may also help to develop and foster the therapeutic alliance (Clough and Casey, 2011). The therapeutic alliance, a common factor in psychotherapy, is supposed to be an essential factor in its outcomes (Mulder et al., 2017). It is characterized by unconditional acceptance, warmth, mutual trust, empathy, shared expectations, beliefs about each other and the therapy, and also safe cooperation on the shared goals of psychotherapy. In the eHealth area, the therapeutic alliance is not a dyadic but a triadic relationship among the users, the e-mental health program, and the program supporter (Cavanagh and Millings, 2013). Some data in the literature indicate that a therapeutic alliance with the e-mental health program can be stated (Ormrod et al., 2010). Although we do not yet know how much the therapeutic alliance matters in e-mental health program effectivity and research and discussion are still in their early stages (Cavanagh and Millings, 2013), everything that supports mental health should be incorporated in eMBPs to maximize their effectivity.

### Aims of the Study

This study aimed to evaluate the feasibility of eMBP, incorporating some of the most effective eHealth program components such as an introductory lecture, a reminder system, text, audio, and video, social support by peers, and lectors in a university setting. We designed an 8-week mindfulness-based program with an innovative intense reminder system incorporating supporting psychotherapy principles (encouraging, advising, reassuring, and self-compassion support) to foster a therapeutic alliance with a team of lectors behind the eMBP and to promote mindfulness practice. In this study, we examined the effect of the program on perceived stress, negative affectivity, self-compassion, quality of life, basic emotion regulation strategies, and mindfulness skills in university students. We hypothesize that participants completing an 8-week eMBP will report significantly lower levels of stress and negative emotional experience and higher levels of mindfulness facets and self-compassion than they report at the start of the intervention. We also hypothesize that the completion of the eMBP will induce a significant change in the frequency use of cognitive reappraisal (higher) and suppression (lower) in the process of emotion regulation in comparison to its measure at the start of the program. In the context of the observed value clarification induced by mindfulness training, it could also be assumed that the importance attributed to the quality of life components could be lower at the end of the intervention than its level at its start.

## Materials and Methods

### Development of eMBP for Stress Reduction in University Students

We developed an 8-week eMBP based on MBCT (Segal et al., 2002). eMBP was run in precise accordance with the content and structure of the book *Mindfulness: A Practical Guide to Finding Peace in a Frantic World* (Williams and Penman, 2011). The program integrates a face-to-face approach (introductory lecture), text, audio, and video components, synchronous social support by peers and lectors, and a unique intensive reminder system supporting formal and informal mindfulness practice and using support psychotherapy principles. eMBP delivery platform used Facebook Messenger in the first run of the program; the Slack messaging application (Slack Technologies, Inc.) replaced Facebook Messenger in the second run. The Slack application allows reminders and weekly programs to be sent automatically; Facebook requires continual personal assistance for sending of reminders. Slack also allows for private conversations between an unlimited number of people; Facebook has a limit of 200 people in an individual group.

The program was divided into three parts: (1) face-to-face introductory lecture, (2) eMBP according to the book program mentioned above, and (3) the reminder system. The introductory lesson lasted 120 min and had eight parts: (1) introduction of the program team, (2) assignment to program run (for ethical and legal reasons, each student had to be personally included in the Facebook and Slack group to prevent anyone outside the university from signing up to the program), (3) motivation section explaining the positive effect of mindfulness on mental and physical health, (4) introduction to the attitudinal foundation of mindfulness practice (Kabat-Zinn, 2013a, p. 21–30), (5) focus on the importance of commitment, self-discipline, intentionality, and personal vision for adherence to the program, (6) introduction to formal and informal mindfulness practice, (7) introduction of the program structure and delivery modes, and (8) discussion.

The course was organized into eight modules. Each week’s module started on Monday morning with a pdf file sent through the mobile application that described the program plan for the whole week. The first week started on Tuesday because of the introductory lecture on Monday. The pdf files ranged from three to seven pages. The program structure was based on Williams and Penman (2011, chapter 4); the structure is described in Supplementary Appendix A. The participants were asked to practice mindfulness formally and informally 6 days of the week. The 1-day retreat was not included in the eMBP.

The reminder system consisted of short messages delivered throughout the day to promote the everyday formal and informal practice. The specific messages were created by the authors of this study based on their clinical experience and on the books *The Mindfulness Solution: Everyday Practices for Everyday Problems* (Siegel, 2010) and *Full Catastrophe Living (Revised Edition): Using the Wisdom of Your Body and Mind to Face Stress, Pain, and Illness* (Kabat-Zinn, 2013a). They consisted of encouragements, reminders, incentives, explanations, metaphors, and recommendations for formal and informal mindfulness practice. In total, there were 456 reminders delivered in the program (7–12 reminders per day). The first reminder was always sent at 7:30 a.m. and the last one at 9 p.m. The other reminders were delivered with 1- or 2-h spacing within the day. Examples of the structure and content of the reminders are presented in Table 1.

**TABLE 1:** Reminder system structure and content.

Type of intervention	Intervention examples	Total^a^	Total original^b^
Welcome message	Good morning. Today is the first day of your mindfulness journey. Please read today’s pdf file, “The First Mindfulness Week” which you can find on the news feed right now. You will get a clear idea of what is waiting for you in the first week and where our shared path will take us.	33^*c*^	33
Formal practice reminder	Have you had time yet today to practice the *mindful body and breathe* exercise? It is hard to find time and there is no need to feel guilty if you haven’t done it yet. But try to find 8 min today.	26	24
Informal practice reminder	Maybe you are having lunch, drinking coffee, going somewhere, or waiting for something. Try to be in fully aware contact with this activity. Without judgment, with patience, with curiosity and acceptance of everything that is happening.	33	31
Be in the present moment reminder	Take a moment to stop and look around. Just be fully aware of where you are and whether your mind is in this place.	71	60
Focus on breathing reminder	Take a moment to acknowledge your breath. Inhale and exhale, nothing more. Do not rush and do not try to change; just acknowledge it fully.	53	31
Focus on body sensations reminder	Take a few moments to realize what feelings you feel in your body. Be aware of where your clothes are touching your body. If you are standing, be aware of the feelings of your legs and feet. If you are sitting, note the sensations at the point where your body touches the chair.	24	20
Focus on sensory inputs reminder	Look around you. Take a moment to be aware of what you see, what you hear, what you smell, the taste in your mouth.	17	5
Observe thoughts and feelings reminder	Stop for a little while and notice from moment to moment what is happening, how your thoughts and feelings appear and how they disappear.	29	25
Basic attitudes reminder	Patience means to be open to every moment, to receive it in its fullness, and to know that things can only take place in their own time. Give this principle 2 min today.	22	20
Personal vision reminder	Maybe you are not following the instructions. Maybe the instructions are just frustrating and upsetting. Maybe you wonder what it’s all about. Try to return to your vision before going to bed, remember why you are in the program and what is important to you.	6	4
Mindfulness metaphors	In the ocean, at a depth of three to six meters, only subtle waves and tranquility are felt, even when there is a great storm on the surface. It is similar when we focus on breathing in the belly. We perceive an area of the body that is far from the head, and thus far below the surface of our raging mind.	9	9
Mindfulness education	The mindfulness exercise begins with focusing attention on breathing and continues on to body sensations, feelings, thoughts, and ultimately comes the experience of self. Gradually, we react increasingly less reactively to everything that appears and gain a greater sense of freedom. Instead of running away from difficult emotions, we are increasingly able to cope with any reaction.	59	56
Time, sleep, work, and study stress	Sometimes it is difficult to sleep in the evening because we cannot get rid of some thoughts. The more we try, the worse it is. Letting things and thoughts be, letting them go at the right time is an important life skill. Remember the principle “let it go.”	11	11
Compassion and gratitude reminder	Try to say to yourself: *May I be free from suffering. May I be as happy and healthy as it is possible for me to be. May I have ease of being. May I learn to not be stuck in the past. May I be able to accept everything that life brings to me.* Whatever you eat today, try to acknowledge where the food comes from, the laws of nature and the people to whom we owe gratitude.	10	9
Self-compassion reminder	In this moment, let yourself be as you are.	27	23
Change in perspective on the self-reminder	Often we are unhappy because we cling to a particular image of ourselves. But if everything is transient, no picture actually exists. So why bother with something that is not real?	9	9
Summary at the end of the day	The evening is a time of peace, a return from the outside world to the inner realm. It is also a time when we often admit many more things that are troublesome to us. Be sensitive and note how many past or future concerns there are. Try the *three-minute breathing space* exercise. Let yourself be led by Mark Williams or do it by yourself, if you know it now. Good night.	17^*c*^	17

**^*a*^Total number of reminders. ^*b*^Total number of original reminders that were not repeated. ^*c*^This number does not match the total number of days in the entire program. It would otherwise be 48 days. Some welcome messages, however, included more than just greetings and so they were included in other type of intervention subgroups.**

The students were encouraged to download audio recordings recommended in the program sourcebook (Williams and Penman, 2011) at the “Mindfulness and Meditation Downloads” website (Random House, 2020), where each recommended meditation is freely available (Meditation 1, Mindfulness of Body and Breath; Meditation 2, The Body Scan; Meditation 3, Mindful Movement; Meditation 4, Breath and Body; Meditation 5, Sounds and Thoughts; Meditation 6, Exploring Difficulty; Meditation 7, Befriending; and Meditation 8, The Three Minute Breathing Space). The foundations of mindfulness practice and its basic attitudes were presented *via* short videos (from 2 min 19 s to 4 min 14 s) on YouTube.com, with each attitude presented by Jon Kabat-Zinn (2015).

Moreover, the students had the opportunity to share their experiences and questions with others within the Facebook group or separate Slack channel, respectively. Sharing was repeatedly encouraged *via* reminders aimed at helping the participants get social support from the group. The participants also had the opportunity to exchange messages with the lecture team regarding any questions or difficulties with formal and informal practice. Participation in the program was completely free.

### Participants

All subjects were students at Masaryk University recruited through advertisements on the website and the Facebook page of the Department of Psychology and Psychosomatics of the Faculty of Medicine. The advertisement was also posted on the web news portal of the university 1 month before the program started. The opportunity to participate in the program was also announced during lectures at the department and on notice boards at the university faculties. The inclusion criterion was that the participants were students at Masaryk University. No exclusion criteria were applied.

In total, 227 students participated in the first run of the program delivered through Facebook messenger (“Facebook group,” mean age 22.3 ± 2.1, 82% women), and 465 students participated in the second run of the program delivered through Slack (“Slack group,” mean age 23.3 ± 2.9, 81% women). The Facebook group program ran in the period between March and May 2018, and the Slack group program ran in the period between October and December 2018. The participants were not randomly assigned to the Facebook and Slack groups.

Both samples consisted of students of medicine (40.4%), humanities (35.8%), and natural sciences (23.8%). No differences in measured variables such as age, gender, and questionnaires were found among these subgroups.

The university ethics committee approved the study (application number 18/2017), and all participants signed informed consent forms at the introductory lecture at the start of the program. Every participant got two matching stickers with a unique number. One sticker was put on the informed consent form and outcome measure questionnaires, and the participant kept the other. This unique code was used as the control measure at the end of the program in a Google Forms version of the outcome measures to anonymize data.

### Outcome Measures

The participants completed the outcome measures questionnaires before the introductory lecture (pen-and-pencil method). The control measurement at the end of the program was created in Google Forms. The link to the questionnaires was sent individually through email to each participant. The notification was sent three times, with 3-day spacing between each notification. Mindfulness, negative and positive affectivity, perceived stress reactivity, self-compassion, emotion regulation, and quality of life were assessed as outcome measures using the following questionnaires.

The Five Facet Mindfulness Questionnaire (FFMQ; Baer et al., 2008; the Czech version, Kořínek et al., 2019) is a widely used tool that measures mindfulness. The questionnaire has five subscales representing the specific mindfulness facets: Describe, Observe, Act With Awareness, Non-judging of Inner Experience, and Non-reactivity to Inner Experience. The items are rated from 1 = “never or very rarely true” to 5 = “very often or always true.” Higher scores indicate higher mindfulness. All items from the Act With Awareness and Non-judge subscales and half of the items in the Describe subscale are reverse-worded. The internal consistency of the subscales ranges between Cronbach’s alpha (Cα) = 0.69 and 0.83; total = 0.77 (Kořínek et al., 2019). The FFMQ scale showed good internal consistency at baseline in this sample (Cronbach’s alpha = 0.88).

The Subjective Emotional Balance Questionnaire (SEBQ; Kožený, 1993) assesses prevailing positive and negative emotional experiencing. The questionnaire has two subscales: Positive Emotional Experiences (e.g., I felt calm and relaxed; I was cheerful) and Negative Emotional Experiences (e.g., I was in a bad mood; I was unhappy), with 18 items in each subscale. The items are evaluated on a scale from 1 = “almost never” to 5 = “very often.” Higher scores indicate higher positive or negative emotional experiencing. The internal consistency was found to be Cα = 0.93 for both scales (Kožený, 1993). The SEBQ scale showed good internal consistency at baseline in this sample (Cronbach’s alpha: negative experiencing = 0.93, positive experiencing = 0.95).

The Perceived Stress Reactivity Scale (PSRS; Schlotz et al., 2011) is a questionnaire assessing typical individual reactivity to everyday life stressors. The scale was translated into Czech by the authors of this study. The scale has 23 items (with 12 items reverse-worded) and five subscales: Prolonged Reactivity (four items), Reactivity to Work Overload (five items), Reactivity to Social Conflict (five items), Reactivity to Failure (four items), and Reactivity to Social Evaluation (five items). The items are rated from 0 = “low-stress reactivity” to 2 = “high-stress reactivity.” The PSRS scale showed good internal consistency at baseline in this sample (Cronbach’s alpha = 0.85).

The Self-Compassion Scale (SCS-SF; Raes et al., 2011; the Czech version, Benda and Reichová, 2016) measures individual compassion for oneself. The SCS-SF is a short form of the 26-item Self-Compassion Scale (SCS) and has a high correlation with the full SCS (*r* ≥ 0.97; Neff, 2003). The items for the short form of SCS were chosen from the standardized and validated Czech version (Benda and Reichová, 2016). The SCS-SF has 12 items and six subscales, with two items in each subscale: Self-Kindness, Self-Judgment, Common Humanity, Isolation, Mindfulness, and Over-identified. The items are rated from 1 = “never” to 5 = “always.” The internal consistency of the subscales ranges between Cα = 0.65 and 0.86; total 0.89 (Benda and Reichová, 2016).

The Emotion Regulation Questionnaire (ERQ; Gross and John, 2003; the Czech version, Marsova, 2016) measures individual differences in the habitual use of two types of emotion regulation strategies: cognitive reappraisal and expressive suppression. The ERQ consists of 10 questions. The items are rated from 1 = “strongly disagree” to 7 = “strongly agree.” The ERQ scale showed good internal consistency at baseline in this sample (Cronbach’s alpha: suppression = 0.86, reappraisal 0.67).

The Subjective Quality of Life Analysis (SQUALA; Zannotti and Pringuey, 1992; the Czech version, Chrastina et al., 2014) measures the quality of life defined as a difference between importance and satisfaction. The tool has 23 areas mapping both internal and external factors affecting everyday life. The questionnaire has two parts: rating of importance and rating of satisfaction in the given areas. The respondents assess both on a scale from 1 = “very satisfied” to 5 = “very disappointed.” The internal consistency was found to be between Cα = 0.82 and 0.90 (Chrastina et al., 2014).

### Data Analysis

Data analysis was conducted with IBM SPSS, version 25, and Jamovi 1.1.0. A dropout analysis by 2 (Facebook group vs. Slack group) × 2 (dropout vs. completed) ANOVAs was calculated for both groups to ensure that dropouts did not differ from completers in baseline outcome measures, gender, or age. The effect of the program was analyzed by 2 × 2 repeated-measures ANOVAs with Bonferroni *post hoc* tests with program as a within-subject variable (before vs. after the program) and group as a between-subject variable (Facebook group vs. Slack group). The Pearson correlation analysis was utilized to determine the relationships between the variables.

## Results

### Dropout

The dropout rate was 97 (43.7%) students (22 men, 75 women, mean age 22.4 ± 2.2) in the Facebook group and 262 (56.3%) students (55 men, 207 women, mean age 22.98 ± 2.7) in the Slack group. These subjects dropped out during the program or did not complete the post-program test battery (program-dropout group).

The 2 (Facebook group vs. Slack group) × 2 (dropout vs. completed) ANOVA was performed to check for possible initial differences between the participants who completed the program and those who dropped out. For this analysis, we looked at whether the dropout factor effect (alone or in interaction) was significant; differences between group 1 and group 2 were not examined. No significant effects of dropout were found in the questionnaires, age, or gender.

### Program Effectivity Analysis

The descriptive statistics of all questionnaire scores are displayed in Table 2. Only participants who completed the program are included in this table. The distribution of all scales was normal. The effect of the program was analyzed by 2 × 2 repeated-measures ANOVA with Bonferroni *post hoc* tests with program as the within-subject variable (before vs. after the program) and group as the between-subject variable (Facebook group vs. Slack group). The results are displayed in Table 3.

**TABLE 2:** Descriptive statistics of dependent variables in both groups before and after eMBP.

Variables	Group 1						Group 2					
		
	Before			After			Before			After		
				
	*N*	*M*	*SD*	*N*	*M*	*SD*	*N*	*M*	*SD*	*N*	*M*	*SD*
PSRS	130	52.81	6.96	130	47.26	7.41	203	53.85	6.87	203	48.72	7.91
SEBQ^a^	130	91.80	22.98	130	79.62	21.23	203	99.92	22.92	203	84.11	21.53
SCS-SF	130	34.31	7.59	130	38.45	7.82	203	33.53	7.03	203	37.64	6.97
FFMQ describing	130	24.36	6.58	130	27.15	5.94	203	24.38	6.74	203	27.06	6.41
FFMQ observing	130	25.51	5.83	130	28.42	4.59	203	24.02	6.07	203	27.98	4.96
FFMQ acting aware	130	22.22	5.49	130	25.45	5.54	203	21.47	5.24	203	24.31	5.05
FFMQ non-judging	130	24.85	6.87	130	28.53	6.45	203	23.36	6.71	203	27.98	6.68
FFMQ non-reacting	130	18.75	4.46	130	21.33	4.11	203	18.07	4.24	203	20.77	4.18
ERQ reappraisal	130	26.61	6.44	130	27.95	6.20	203	25.86	6.99	203	27.99	6.05
ERQ suppression	130	13.06	4.36	130	12.62	3.83	203	13.23	3.51	203	13.16	3.70
SQUALA satisfaction	130	47.92	7.66	130	47.45	9.25	203	45.41	8.23	203	45.41	8.04
SQUALA importance	130	57.04	9.90	130	51.88	11.37	203	57.77	10.73	203	52.32	10.94

**^*a*^Higher values indicate more negative experiences; PSRS, The Perceived Stress Reactivity Scale; SEBQ, The Subjective Emotional Balance Questionnaire; SCS-SF, The Self-Compassion Scale-Short Form; FFMQ, The Five Facet Mindfulness Questionnaire; ERQ, The Emotion Regulation Questionnaire; SQUALA, Subjective Quality of Life Analysis.**

**TABLE 3:** The effects of program and group and interaction effects for ANOVA.

Variables	Program effect			Group effect			Program*Group effect			Bonferroni *post hoc* tests
			
	*F*(1, 331)	p	_*p*_η^2^	*F*(1, 331)	*p*	_*p*_η^2^	*F*(1, 331)	*p*	_*p*_η^2^	
PSRS	**237.394**	**< 0.001**	**0.418**	2.814	0.094	0.008	0.355	0.552	0.001	*Program change in group 1 t*(331.000) = 10.249, *p* < 0.001 *Program change in group 2 t*(331.000) = 11.853, *p* < 0.001
SEBQ	**151.547**	**< 0.001**	**0.314**	**8.081**	**0.005**	**0.024**	2.551	0.111	0.008	*Program change in group 1 t*(331.000) = 6.861, *p* < 0.001 *Program change in group 2 t*(331.000) = 11.129, *p* < 0.001 *Group difference before program t*(437.941) = −3.258, *p* = 0.007 *Group difference after the program t*(437.941) = −1.801, *p* = 0.433
SCS-SF	**126.857**	**< 0.001**	**0.277**	1.168	0.281	0.004	0.002	0.967	0.000	*Program change in group 1 t*(331.000) = −7.239, *p* < 0.001 *Program change in group 2 t*(331.000) = −8.980, *p* < 0.001
FFMQ describing	**87.047**	**< 0.001**	**0.208**	0.003	0.960	0.000	0.037	0.848	0.000	*Program change in group 1 t*(331.000) = −6.098, *p* < 0.001 *Program change in group 2 t*(331.000) = −7.313, *p* < 0.001
FFMQ observing	**126.749**	**< 0.001**	**0.277**	3.361	0.068	0.010	2.905	0.089	0.009	*Program change in group 1 t*(331.000) = −6.118, *p* < 0.001 *Program change in group 2 t*(331.000) = −10.373, *p* < 0.001
FFMQ acting aware	**107.820**	**< 0.001**	**0.246**	3.342	0.068	0.010	0.453	0.501	0.001	*Program change in group 1 t*(331.000) = −7.081, *p* < 0.001 *Program change in group 2 t*(331.000) = −7.771, *p* < 0.001
FFMQ non-judging	**127.041**	**< 0.001**	**0.277**	2.465	0.117	0.007	1.643	0.201	0.005	*Program change in group 1 t*(331.000) = −6.397, *p* < 0.001 *Program change in group 2 t*(331.000) = −10.046, *p* < 0.001
FFMQ non-reacting	**129.376**	**< 0.001**	**0.281**	2.203	0.139	0.007	0.066	0.797	0.000	*Program change in group 1 t*(331.000) = −7.119, *p* < 0.001 *Program change in group 2 t*(331.000) = −9.308, *p* < 0.001
ERQ reappraisal	**26.000**	**< 0.001**	**0.073**	0.300	0.584	0.001	1.349	0.246	0.004	*Program change in group 1 t*(331.000) = −2.522, *p* = 0.073 *Program change in group 2 t*(331.000) = −5.010, *p* < 0.001
ERQ suppression	1.250	0.264	0.004	0.971	0.325	0.003	0.701	0.403	0.002	–
SQUALA satisfaction	0.447	0.504	0.001	**6.962**	**0.009**	**0.021**	0.447	0.504	0.001	*Group difference before program t*(437.941) = 2.696, *p* = 0.044 *Group difference after the program t*(437.941) = 2.191, *p* = 0.174
SQUALA importance	**119.780**	**< 0.001**	**0.266**	0.280	0.597	0.001	0.093	0.760	0.000	*Program change in group 1 t*(331.000) = 6.813, *p* < 0.001 *Program change in group 2 t*(331.000) = 9.003, *p* < 0.001

**PSRS, The Perceived Stress Reactivity Scale; SEBQ, The Subjective Emotional Balance Questionnaire; SCS-SF, The Self-Compassion Scale-Short Form; FFMQ, The Five Facet Mindfulness Questionnaire; ERQ, The Emotion Regulation Questionnaire; SQUALA, Subjective Quality of Life Analysis. Bold values indicate the statistically significant result.**

The pre- and post-program comparison in both experimental groups showed that the eMBP led to a decrease of the perceived stress reactivity (see pη^2^ in Table 2), an increase of positive and decrease of negative emotional experiencing, an increase in subjective mindful experiencing in each measured facet, and an increase in self-compassion. Furthermore, the comparisons in both groups revealed that the eMBP led to an increase in the use of cognitive reappraisal as an emotion regulation strategy. In contrast, the use of suppression was not affected by the program. Finally, the importance attributed to the quality of life components decreased after the program, while satisfaction with life remained unchanged.

Besides the significant effect of program, a significant effect of group was found in the subjective emotional balance, driven by a significant difference between the groups before the program, but not after the program, while in both groups negative affectivity (SEBQ) decreased significantly. A significant effect of group only was found in satisfaction with life (SQUALA), driven by a borderline significant difference in satisfaction between the groups before the program, but not after the program. However, none of the groups improved significantly in satisfaction after the program.

### Correlation Analysis

The correlation analysis revealed a significant negative association between mindfulness as measured by all subscales of FFMQ and perceived stress and prevailing positive and negative emotional experiencing at the start of the program (see Table 4). The higher the level of mindfulness experienced, the less that stress and negative emotivity experiences are reported; positive emotivity experience increases. The same trend of association, but a stronger one, was found between self-compassion, stress, and prevailing positive and negative emotional experiencing. Correlations show that the higher the level of reported self-compassion, the less stress and negative emotivity are experienced.

**TABLE 4:** Correlations between dependent variables before the eMBP.

	1	2	3	4	5	6	7	8	9	10	11	12
1. FFMQ describing	–	0.27***	0.27***	0.22***	0.30***	−0.36***	0.30***	−0.21***	0.22***	−0.25***	−0.08*	−0.17***
2. FFMQ observing		–	0.13***	0.00	0.34***	−0.14***	0.25***	−0.19***	0.29***	−0.08*	−0.09*	−0.08*
3. FFMQ acting aware			–	0.37***	0.35***	−0.37***	0.31***	−0.27***	0.12**	−0.09*	0.01	−0.24***
4. FFMQ non-judging				–	0.41***	−0.48***	0.56***	−0.44***	0.18***	−0.20***	0.02	−0.33***
5. FFMQ non-reacting					–	−0.55***	0.61***	−0.40***	0.41***	0.00	−0.05	−0.22***
6. PSRS						–	−0.63***	0.47***	−0.31***	0.11**	−0.07	0.33***
7. SCS-SF							–	−0.52***	0.49***	−0.12**	−0.06	−0.36***
8. SEBQ^*a*^								–	−0.40***	0.22***	0.12**	0.57***
9. ERQ reappraisal									–	−0.07	−0.16***	−0.29***
10. ERQ suppression										–	0.13***	0.19***
11. SQ satisfaction											–	0.20***
12. SQ importance												–

^*a*^Higher values indicate more negative experiences; PSRS, The Perceived Stress Reactivity Scale; SEBQ, The Subjective Emotional Balance Questionnaire; SCS-SF, The Self-Compassion Scale-Short Form; FFMQ, The Five Facet Mindfulness Questionnaire; ERQ, The Emotion Regulation Questionnaire; SQ, Subjective Quality of Life Analysis; *p < 0.05, **p < 0.01, ***p < 0.001.

Interestingly, a significant correlation was found between the FFMQ subscales Acting Aware, Non-judging and Non-reacting, and Importance attributed to the quality of life components (SQUALA importance subscale). The association between these variables is even closer at the end of the eMBP (Table 5). Self-compassion is also negatively associated (at a medium level) with the importance attributed to the quality of life components at the start and the end of the program (Tables 4, 5).

**TABLE 5:** Correlations between dependent variables after the eMBP.

	1	2	3	4	5	6	7	8	9	10	11	12
1. FFMQ describing	–	0.37***	0.35***	0.34***	0.39***	−0.39***	0.34***	−0.30***	0.15**	−0.31***	−0.09	−0.26***
2. FFMQ observing		–	0.36***	0.28***	0.46***	−0.28***	0.35***	−0.38***	0.29***	−0.26***	−0.14*	−0.26***
3. FFMQ acting aware			–	0.50***	0.43***	−0.45***	0.42***	−0.42***	0.18**	−0.19***	−0.02	−0.25***
4. FFMQ non-judging				–	0.48***	−0.47***	0.58***	−0.55***	0.26***	−0.17**	−0.04	−0.28***
5. FFMQ non-reacting					–	−0.60	0.61***	−0.48***	0.36***	−0.11*	−0.11*	−0.31***
6. PSRS						–	−0.69***	0.56***	−0.33***	0.10	0.11*	0.41***
7. SCS-SF							–	−0.62***	0.44***	−0.13*	−0.15**	−0.38***
8. SEBQ^a^								–	−0.43***	0.19***	0.16**	0.61***
9. ERQ reappraisal									–	−0.06	−0.19***	−0.26***
10. ERQ suppression										–	0.09	0.14**
11. SQ satisfaction											–	0.34***
12. SQ importance												–

^*a*^Higher values indicate more negative experiences; PSRS, The Perceived Stress Reactivity Scale; SEBQ, The Subjective Emotional Balance Questionnaire; SCS-SF, The Self-Compassion Scale-Short Form; FFMQ, The Five Facet Mindfulness Questionnaire; ERQ, The Emotion Regulation Questionnaire; SQ, Subjective Quality of Life Analysis; *p < 0.05, **p < 0.01, ***p < 0.001.

The analysis also revealed a significant positive association (small to medium) between reported mindfulness experiencing and cognitive reappraisal and a negative association with suppression before and after the program. At the same time, this association is stronger for suppression at the end of the program (Table 5). A significant positive association can also be observed between cognitive reappraisal (medium) and negative with suppression (low).

Cognitive reappraisal is associated with positive emotivity, and suppression is related to negative emotivity (Tables 4, 5).

### The Component Adherence Analysis

The control measure at the end of the eMBP in the Facebook and Slack groups contained some partial questions about adherence to the program. Table 6 presents self-reported adherence measures. The results show that 3.1% of students never and 28.3% rarely finish the task at the time when it was sent *via* the messaging application (answer 2 in Table 6). It further showed that 4.4% of students never and 22.1% once managed to do the exercises recommended for the concrete week. Regardless, they filled out the questionnaires at the end of the program. Answers for question 5 show that an irregular pattern of formal mindfulness practice is prevalent in 73.4% of students. Some negative effect of mindfulness practice was reported by 1.2% of students. Self-reported data also show (questions 7 and 8; Table 6) that a higher rate of students completed the program at the level of the reminder system (53.4%) than the recommended formal practice for each week (38.6%).

**TABLE 6:** The self-reported parameters of adherence to eMBP (*n* = 333).

Always	Very often	Sometimes	Rarely	Never	
*1. Were you able to read the reminders at the time of delivery?*					
7.5%	37.8%	29.8%	22.1%	2.8%	
*2. Were you be able to finish the task in time when it was sent via the messenging app?*					
2.7%	17.4%	48.5%	28.3%	3.1%	
*3. Were you be able to read the pdf file each week of the program?*					
32%	22.4%	21.1%	19.5%	5%	
*4. How often did you manage to do the exercises for the week?*					
Exactly as recommended	Exactly as recommended, but just a few times a week	Just some exercises, but every day	Just some exercises, but not every day	I tried it once, but I did not continue	I never tried any
0.6%	5%	15.2%	52.8%	22.1%	4.4%
*5. When did you most often practice mindfulness during the day?*					
In the morning and evening	In the morning	In the evening	Irregularly during the day when I had time		
7.6%	4.7%	14.3%	73.4%		
*6. Mindfulness practice in eMBP has influenced me:*					
It has not influenced me in any way	It has influenced me negatively	It has influenced me positively			
18.3%	1.2%	80.5%			
*7. What percent of the program do you think you completed (reminder system)?*					
53.4 ± 25.3					
*8. What percent of the program do you think you completed (formal practice)?*					
38.6 ± 22.5					
*9. How much do you believe that you will be able to practice mindfulness in your life after the program ends? (1* = *“I will not continue” to 10* = *“I will continue”)*					
6.1 ± 2.5					

At the end of the data collection in the Slack group, a short version (three close questions and one open question) survey was sent to the dropout group to get information about how long they followed the program and if they finished it. One hundred sixty-one students from the dropout group answered. One hundred fourteen of them did not complete the eMBP. The average time of the attrition rate was 2.78 ± 1.62 weeks (minimum, 0; maximum, 7). Even though they dropped out of the program, 46 of them reported that eMBP had influenced their life positively (45 reported no influence, and the rest of them did not respond).

Forty-seven students of the 161 who dropped out and answered the short version of the survey completed the program, but they did not have enough time or will to complete the final battery of questionnaires. The open question concerned the reasons for dropout. The reasons of the 161 drop-out students were very heterogeneous, and there were usually combinations of several reasons. The most prevalent reasons were as follows: (1) lack of time for formal and informal practice (56.7%), “If I can’t do it for one hundred percent, I won’t do it at all”, (2) realizing that the program was not suitable for them in its content or form (7.2%), (3) loss of motivation (42.3%), (4) dissatisfaction with Facebook or Slack that requires being online (10.3%; they would prefer offline app), and (5) too intensive reminder system (17.2%; they would prefer about four messages per day). These respondents often explained that the reminder system was not too intense in its content. They appreciated it; however, the higher frequency of the reminders reminded them that they were not fulfilling the program tasks according to the recommendations, and it was stressful for them.

A correlation analysis was performed to find any evidence of a relationship between the self-reported percentage of adherence to the eMBP formal and informal practices and the positive psychological outcomes. Small significant correlations, presented in Table 7, partially imply that the more students were adhering to the program, the more they experienced some aspects of a mindful approach to reality, self-compassion, and increased use of cognitive reappraisal; they experienced less stress and negative emotivity.

**TABLE 7:** Correlations between dependent variables and the self-reported percentage of adherence to the eMBP formal and informal practice.

	Self-reported % of informal practice completed (reminder system; question 7 in Table 6)	Self-reported % of formal practice completed (whole program; question 8 in Table 6)
1. FFMQ describing	0.24**	0.20**
2. FFMQ observing	0.28**	0.32**
3. FFMQ acting aware	0.10	0.10
4. FFMQ non-judging	0.20**	0.12*
5. FFMQ non-reacting	0.22**	0.24**
6. PSRS	−0.10	−0.19**
7. SCS-SF	0.13*	0.22**
8. SEBQ^*a*^	−0.23**	−0.22**
9. ERQ reappraisal	0.20**	0.26**
10. ERQ suppression	−0.04	0.05
11. SQ satisfaction	0.04	−0.07
12. SQ importance	−0.08	−0.09

^*a*^Higher values indicate more negative experiences; PSRS, The Perceived Stress Reactivity Scale; SEBQ, The Subjective Emotional Balance Questionnaire; SCS-SF, The Self-Compassion Scale-Short Form; FFMQ, The Five Facet Mindfulness Questionnaire; ERQ, The Emotion Regulation Questionnaire; SQ, Subjective Quality of Life Analysis; *p < 0.05, **p < 0.001.

**FIGURE 1:**
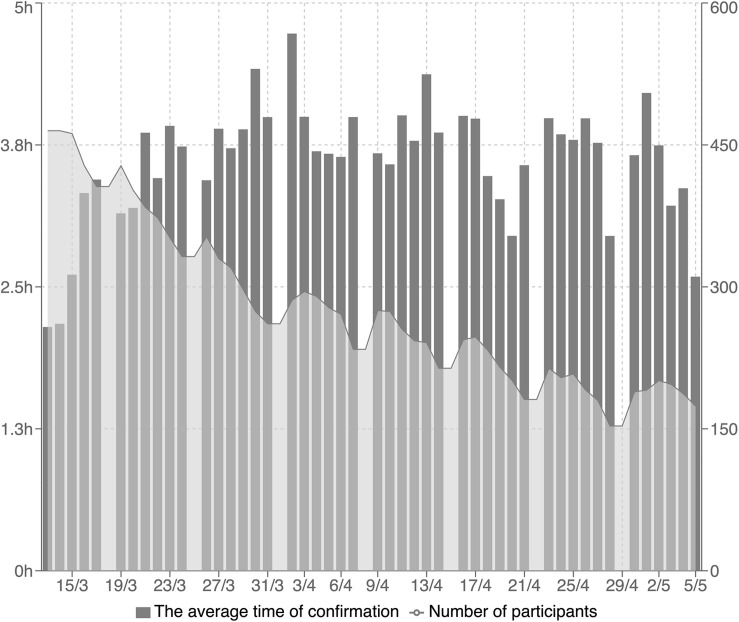
The average time of reactions to reminders (gray columns) and the total number of participants who confirmed all the daily reminders on each particular day (gray line). In this figure shows the average time of reactions to reminders (gray columns; each column represents 1 day in the program from Monday to Saturday). Only the students who confirmed reading all the reminders on each particular day were included in this analysis. The gray line in the figure represents the total number of participants who confirmed reading all the daily reminders each day.

In the partial adherence analysis in the Slack group (Facebook manager does not allow this analysis), we were interested in how many students react to each reminder on different days, what the average reaction time to it was, and the total number of participants who confirmed all the daily reminders on each particular day (Figure 1). Each reminder had a small green checkmark below it. The students were instructed to check it as soon as possible after reading each reminder.

## Discussion

### Program Effectivity Analysis

The pre–post completer analysis indicates the large eMBP intervention effect on the decrease of perceived stress and a medium effect on affect experience, self-compassion, and mindful approach to the entire experience. These results were observed in both runs of the program repeatedly. The effect sizes detected in our study are comparable with the mean results of other studies conducted on university students using mindfulness-based interventions and participants from the non-clinical population in eHealth version (Krusche et al., 2012; Cavanagh et al., 2018; Danilewitz et al., 2018; Querstret et al., 2018) and in face-to-face programs as well (Rosenzweig et al., 2003; Lynch et al., 2011; Warnecke et al., 2011; Barnes et al., 2017). This partial comparability of the eMBP to other mindfulness therapies delivered face to face is hopeful. It corresponds with the challenge to find a useful program for mental health support for a broad group of students. It also shows the meaningfulness of further initiatives in this area. The increasing interest of students in completing the eMBP at our university (first run 227 students and second run 465 students) also shows that the eHealth mode of delivery is feasible for them.

Our study presents the finding that students who underwent the eMBP significantly increased the use of cognitive reappraisal with a small effect size. Although increased emotion regulation induced by mindfulness probably involves a mix of the many implicit and explicit regulation strategies and processes comprehensively described by Gross (2014), this corresponds with previous suggestions and results that cognitive reappraisal seems to be one of the core emotion regulation strategies during mindfulness training (Feldman et al., 2007; Garland et al., 2011; Hölzel et al., 2011). Garland et al. (2011) documented that mindful emotion regulation works through positive reappraisal. Under mindfulness practice, stress is reinterpreted, such as being beneficial and meaningful. At a higher level of emotion regulation organization, mindfulness can also be understood as cognitive reappraisal at a process level rather than at a content level (Chambers et al., 2009). Through the practice, the meaning of a whole experience (thoughts, emotions, sensations) is cognitively reappraised. In the open monitoring mode of mindfulness practice, no particular aspect of the continuously changing experience is chosen to influence emotions. Attention is paid instead to everything in a non-judgmental and accepting manner. Immediate emotional responses are not regulated; they are simply accepted. Emotional responses are observed with interest and curiosity, becoming objects of observation themselves. This notion supports our partial finding of a moderate significant correlation (*r* = 0.41, Table 3) between the Non-reacting subscale of FFMQ and cognitive reappraisal. Despite the fact that mindfulness seems opposed to expressive suppression (Chambers et al., 2009) and the mindful way of experience processing is associated with less experiential avoidance and thought suppression (Feldman et al., 2007), the expected reduction of suppression was not found in our study. Nevertheless, there was an observable trend in small negative correlations between suppression and FFMQ subscales, which suggests the expected contradiction of suppression and the mindful approach to the entire experience. The measurement of emotion regulation strategy frequency use is still sporadic in the context of eMBP research. However, Cavanagh et al. (2018), for example, documented a decrease of perseverative thinking under a brief eMBP.

We found some supportive data showing that the importance attributed to the quality of life components would be lower at the end of the program, with an unchanged satisfaction with partial components of quality of life proposed in SQUALA. The decrease of importance, with a medium-sized effect, while maintaining unchanged components of life satisfaction, represents a specific shift induced by the program, and it is a new result in this area. These results were replicated in two consecutive runs of the same program. Mindfulness techniques, as part of the “third wave” of cognitive and behavioral therapies, help to target contexts and functions of psychological phenomena, not just their form (Hayes, 2016). Unlike other therapeutic strategies, such as cognitive–behavioral therapy, mindfulness-based interventions do not emphasize changing the contents of mental events as much as changing the awareness of and relationship to them (Segal et al., 2002). The participants learn to disempower emotionally charged thoughts or attitudes by bringing to their experience a sense of “allowing” it to be just as it is, without a constant need for the situation to match their desired states (Segal et al., 2002). It could be suggested that, by staying in the experience of a present moment in a non-reactive and accepting way and by witnessing the impermanence of mental phenomena, the participants learn to strengthen their inner resources and trust the ever-changing conditions of everyday life, instead of constantly relying on external factors to make their life better. This could make them less overly dependent on these factors and therefore mark the factors as less important in terms of life satisfaction. In this context, for instance, Shapiro et al. (2006) proposed that one mechanism of change induced by mindfulness training is value clarification. Mindfulness makes it possible to see clearly what is essential for a satisfying life. This change could also be related to the process of “decentering” (Fresco et al., 2007), defined as disengaging the self from the event, which is commonly reported as an important factor regarding mindfulness mechanisms (Lebois et al., 2015). For example, decentering mediated a decrease in anxiety in an MBSR program with university students (Fresco et al., 2007). We could hypothesize that a decrease of importance could be a specific marker of mindfulness-induced change, and it could be a co-mediator between mindfulness and its positive outcomes. Interestingly, the significant negative correlations between the FFMQ subscales Acting Aware, Non-judging and Non-reacting, and Importance that were even more closely attributed to the quality of life components at the end of the eMBP indirectly open this explanation.

### Dropout and Adherence

Of the 227 subjects who participated in the first run of the program and the 465 who participated in the second run, 51.95% (43.7% first run, 56.3% second run) completed the eMBP in that they completed the post-program test battery. This rate of attrition corresponds approximately to the findings of similar studies. Cavanagh et al. (2013) reported that 52.3% of web-based study participants completed the questionnaires at pre- and post-intervention. The same research group documented that 68% completed questionnaires at pre- and post-intervention in another study (Cavanagh et al., 2018). Forbes et al. (2018) presented similar results (53.3%). Howells et al. (2016) used a smartphone app to deliver mindfulness training and reported a 77% dropout rate. A dropout rate of 25% was described by Querstret et al. (2018) in their 4-week mindfulness online program. The 10% attrition cutoff is recommended by the Cochrane risk of bias tool (Higgins et al., 2011). In this background, it seems quite conservative in eHealth interventions as higher attrition rates are usually reported. The optimal attrition cutoff level is still waiting for the specification in this field.

Thanks to the short-version survey addressed to the dropout group (359 students in sum), we obtained additional partial information from 161 of them. We found out that still 47 students completed the program without completing the final questionnaire. In this context, the attrition rate is a little bit lower (45.1% in sum). One possible explanation is that the program ends before the exam period, and many students may have decided that they did not have time for it. This finding documents that there is a discrepancy in some subgroups of participants between adherence to the program and the motivation to complete a relatively time-consuming test battery. The common methodological problem is that programs are effective for people who stayed in them (Van Dam et al., 2018), but who are those who dropped out? In this context, one of the fundamental characteristics and methodical challenges in the evaluation of eHealth and mHealth applications is thus the phenomenon of participants stopping usage and/or being lost to follow-up, termed as the law of attrition by Eysenbach (2005). He argued that non-usage data themselves should be of great interest to researchers, as describing patterns and predictors for attrition and non-adherence research offers much information about treatment itself as well as data on system usability. According to his proposal, attrition can be split into two different processes: dropout attrition or the phenomenon of losing participants to follow-up (47 students completed the program but did not fill out the survey) and non-usage attrition or the proportion of participants who do not drop out (e.g., they still fill in questionnaires) but who are no longer following the program. They are non-adherent, in other words. As can be seen in Table 6 (questions 1–4), almost 30% of the participants reported that they never or rarely followed the formal and informal parts of the program, and they still completed the final survey.

According to a partial analysis of reactions to reminders in the Slack group (Figure 1), the steepest dropout in the sense of response to reminders on time was during the first 2 or 3 weeks. Then, it decreased slowly. Unfortunately, we do not know if the dropout from the reminder system also meant a dropout from the program itself in the sense of recommendations from the pdf file for each week. This trend is similar as what was observed in another study (Forbes et al., 2018). A higher dropout rate during the first 3 weeks of an Internet-based 8-week mindfulness program was also reported by Kvillemo et al. (2016). It also indeed indirectly documents the finding that 114 students did drop out on average during the first 3 weeks (2.78 ± 1.62; minimum, 0; maximum, 7). Even though they dropped out of the program, 46 of them reported that eMBP had influenced their life positively. If we use MBCT or MBSR programs as a golden standard in this intervention area, there is some consensus among experts, also supported with some experimental data, that 4-week mindfulness programs seem to be efficacious for promoting well-being and stress reduction, and this length of completion can be considered as a minimum adequate “dose” (Demarzo et al., 2017; Crane and Hecht, 2018). The feasibility and effectiveness of shorter online self-guided mindfulness-based interventions have been demonstrated (Cavanagh et al., 2013). The question of what is enough (length and content) for the eMBP positive effect on mental health in the eHealth area is still open.

The partial results from Table 6 (questions 7 and 8) reveal that the students followed the reminder system in higher percentages (53.4 ± 25.3) than they followed the formal practice part of the program (38.6 ± 22.5). There is growing evidence in the literature about positive results associated with reminders (text messages) in a variety of settings of healthcare services (Schwebel and Larimer, 2018). Wells et al. (2020) documented the importance and effectivity of smart messaging reminding oncology patients in an MBCT program of prescribed between-session activities. The program completion was eight times greater for patients using smart messaging compared with non-users. A study comparing intervention arms with and without reminders is still missing in the field of eMBPs generally. In this context, the correlations between dependent variables and the self-reported percentage of adherence to reminders (Table 7) show that they can support the change of attitudes, beliefs, and behavior. Reminders should continue to be evaluated and improved to find out the most effective timing and frequency of messages for improving program outcomes (Schwebel and Larimer, 2018). The effectiveness and attrition rates of our program are comparable with the results of other studies that did not use such an intensive reminder system (Krusche et al., 2012, 2013; Cavanagh et al., 2013, 2018; Querstret et al., 2018). In this context, it is necessary to provide some eMBP to a large sample of students to have an opportunity to manipulate the various variables in different study arms (e.g., reminders vs. without, introductory lecture with the facilitator, online chat, web, app or its combination, reward, etc.).

Previous studies raised expectations that high mindfulness traits at the start of the program would predispose the participants to adherence (Forbes et al., 2018), but our results differed. No significant difference between the program and dropout groups was found in the mindfulness traits measured by FFMQ. This confirms the investigation by Cavanagh et al. (2018) showing no significant differences between participants who completed the study and those who dropped out in the mindfulness baseline. The current study also found that adherent and non-adherent participants did not significantly differ in any of the remaining measured variables. Levels of perceived stress, use of emotion regulation strategies, and subjective emotional balance did not predict who would complete the questionnaires at the post-intervention stage.

Although we did not conduct any mediational model among monitored variables, our results indirectly support that the effect of eMBP on the decrease of stress and negative affect experiencing could be mediated by mindfulness and self-compassion (see Tables 4, 5). Mindfulness and self-compassion are considered to be transtherapeutic and transdiagnostic phenomena that play roles in the development and maintenance of mental health and quality of life (Schanche, 2013; Greeson et al., 2014). The increase of mindful ways of experience processing and self-compassion was found to significantly mediate the effects of eMBP on stress (Gu et al., 2017).

## Values, Limitations, and Future Directions

The results of the presented pilot study confirmed that eMBP is a feasible tool in university students’ mental health support. It revealed that the students who completed the eMBP reported a reduction of perceived stress, a decrease of negative affect experience frequency and intensity (*vice versa* with positive affectivity), an increase of being mindful in their life, and a higher rate of self-compassion. A significant change in the frequency of using some adaptive emotion regulation strategies and feelings of life satisfaction was also observed. Our study provides a new result in the observation of a significant decrease in attributed importance to the quality of life components. The study documented that mindfulness-based interventions can be effectively delivered *via* eHealth form to university students. In our study, we introduced an online eMBP based on MBCT, combining a face-to-face approach (introductory lecture) with text, audio, video, and e-learning components integrated into a unique intensive reminder system using support psychotherapy principles. The study used Facebook as a popular tool for social networking and also the less widespread tool for team communication Slack, which offers many options and benefits for use in eHealth intervention settings.

The pilot study design does not allow us to eliminate the possibility that the positive results of online mindfulness programs could be explained by the fact that everyone who did not benefit dropped out (Forbes et al., 2018). We did not use a randomized wait-list and active control design, so the change cannot be readily attributed to the eMBP rather than to non-specific processes of change (e.g., participant expectation of benefit). The missing comparison of our MBP with similar supporting interventions (online and face to face) also does not allow us to attribute a program effect to this very MBP. The external validity of the study cannot be adequately assessed at this time because of the greater potential for bias in subject selection. We are also aware that high dropout is a risk of bias. Excluding students who do not adhere to the research protocol (did not get their intended content of eMBP) from the analysis can have significant implications that would impact the study’s results and analysis. The most effective way to establish a causal relationship between an intervention and outcome is through a randomized controlled trial (RCT) study design combined with the intention-to-treat analysis (McCoy, 2017). In this context, a recent RCT study by El Morr et al. (2020) has revealed that an 8-week web-based mindfulness intervention for university students effectively reduces common mental health conditions such as depression and anxiety symptoms and in increasing mindful approach to the entire experience. Our pilot study did not include any follow-up control, so we were not able to evaluate the reported induced change over time. We also did not assess any possible mediation effects among variables. We also are not able to separate the effect of the 8-week mindfulness program based on MBCT and the possible simple effect of the intensive reminder system. We have no evidence of how much added value the intensive reminder system *via* Facebook and Slack had.

## Data Availability Statement

The raw data supporting the conclusions of this article will be made available by the authors, without undue reservation, to any qualified researcher.

## Ethics Statement

The study protocol was approved by the ethics committee at the Faculty of Medicine of Masaryk University, Brno, CZ EU (application number 18/2017) and informed consent was obtained from each participant before participation. The patients/participants provided their written informed consent to participate in this study.

## Author Contributions

MS designed and executed the study, prepared the online program, assisted with data analysis, and wrote the manuscript. PL collaborated in analyzing the data, wrote part of the “Results” section and edited the final manuscript. TK, BK, JK, VH, KJ, and KL converted the program to Facebook and Slack and executed the study. AS and RŠ collaborated in the writing and editing of the final manuscript. All authors contributed to the article and approved the submitted version.

## Conflict of Interest

The authors declare that the research was conducted in the absence of any commercial or financial relationships that could be construed as a potential conflict of interest.
