# Supplementary material for: Being Mindful at University: A Pilot Evaluation of the Feasibility of an Online Mindfulness-Based Mental Health Support Program for Students
Source: Front Psychol. 2021 Jan 11;11:581086. doi: 10.3389/fpsyg.2020.581086 (PMC7829670; doi:10.3389/fpsyg.2020.581086)
Supplement: Supplementary file 1 [file Data_Sheet_1.docx]

Appendix. eMBP protocol

An overview of the MBCT protocol used with the specific mindfulness themes of each week is described in detail in Williams and Penman (2011, pp 80).

Week 1: Theme: Waking up to the autopilot

This week is aimed at helping people be more aware of the automatic pilot mode of their minds and encourage them to explore the nonautomatic way of being.

Pdf file (three pages): introduction to the first-week content and structure and description of exercises.

Exercise: Return to the personal vision of why to start, chocolate meditation, mindful awareness of a routine daily activity (e.g., brushing teeth), Mindfulness of the Body and Breath meditation twice a day, six out of seven days (track one at “Mindfulness and Meditation Downloads”), and one breath meditation.

The reminder system (see Table 1): amount 54; contents 4 short videos (the attitudinal foundation of Mindfulness: nun-judging, patience, trust, beginner’s mind; Kabat-Zinn, 2015) and formal and informal practice reminders.

Week 2: Theme: Keeping the body in mind

This week is aimed at helping people to train the focus of attention directly on the bodily sensations without judging and analyzing it to get clear access to the difference between the “thinking mind” and the “sensing mind.”

Pdf file (five pages): recapitulation of the previous week, introduction to the second week content and structure and description of exercises.

Exercise: Body Scan practice twice a day, six out of seven days (track two at Mindfulness and Meditation Downloads), carry out another routine activity mindfully (a different one from last week), a walk for at least fifteen minutes at least once this week, and the ten-finger gratitude exercise.

The reminder system: amount 60; contents 4 short videos (the attitudinal foundation of Mindfulness: nun-judging, non-striving, acceptance; Kabat-Zinn, 2015) and formal and informal practice reminders.

Week 3: Theme: The mouse in the maze

This week is aimed at allowing people, through mindful movement practices based on yoga, see how tense, angry, or unhappy they can become when things do not turn out the way they want.

Pdf file (six pages): recapitulation of the previous week, introduction to the third week content and structure and description of exercises.

Exercise: Eight minutes of Mindful Movement meditation (track three at Mindfulness and Meditation Downloads) followed by an eight-minute Breath and Body meditation (track four), a three-minute Breathing Space meditation, to be practiced twice a day (track eight), and carry out a routine activity mindfully.

The reminder system: amount 56; contents 3 short videos (the attitudinal foundation of Mindfulness: letting go and generosity; Kabat-Zinn, 2015 and “What is Mindfulness” by Kabat-Zinn, 2013b at youtube.com) and formal and informal practice reminders for this and other weeks.

Week 4: Theme: Moving beyond the rumor mill

This week is aimed at teaching people to see their thoughts as mental events that come and go just like sounds and to take a “decentered” stance to thoughts and feelings.

Pdf file (six pages): recapitulation of the previous week, introduction to the fourth week content and structure and description of exercises.

Exercise: An eight-minute Breath and Body meditation (track four), an eight-minute Sounds and Thoughts meditation, to be practiced twice a day (track five), A three-minute Breathing Space meditation (track 8), to be practiced twice a day and also whenever they need it at any other time.

The reminder system: amount 52; contents 4 short videos (the attitudinal foundation of mindfulness: acceptance, non-striving, trust; Kabat-Zinn, 2015 and “5 Ways to be Happy with the Happiest Man on Earth” by Matthieu Ricard (2016) at youtube.com) and formal and informal practice reminders for this and other weeks.

Week 5: Theme: Turning toward difficulties

This week is aimed at helping people to face (rather than avoid) the difficulties that arise in their life from time to time.

Pdf file (seven pages): recapitulation of the previous week, introduction to the fifth week content and structure and description of exercises.

Exercise: An eight-minute Breath and Body meditation (track four), an eight-minute Sounds and Thoughts meditation (track five), a ten-minute Exploring Difficulty meditation (track six), three meditations are practiced in sequence, effectively rolled into one and practiced once each day.

The reminder system: amount 63; contents 1 short video (the attitudinal foundation of mindfulness: acceptance; Kabat-Zinn, 2015) and formal and informal practice reminders for this and other weeks.

Week 6: Trapped in the past or living in the present

This week is aimed at helping people to explore how negative ways of thinking gradually dissipate via the cultivation of loving-kindness and compassion.

Pdf file (five pages): recapitulation of the previous week, introduction to the sixth week content and structure and description of exercises.

Exercise: the ten-minute Befriending meditation (track seven) practiced once each day, and an eight-minute Breath and Body meditation (track four) as preparation for it. The continuation with the three-minute Breathing Space meditation is also recommended twice a day and whenever it is needed.

The reminder system: amount 53; contents 1 short video (the attitudinal foundation of mindfulness: trust; Kabat-Zinn, 2015) and formal and informal practice reminders for this and other weeks.

Week 7: When did you stop dancing

This week is aimed at helping people to explore the close connection among our daily routines, activities, behavior, and emotions, and to be aware that stress and exhaustion usually lead to giving up activities and things that “nourish” them.

Pdf file (four pages): recapitulation of the previous week, introduction to the seventh week content and structure and description of exercises.

Exercise: it is suggested that participants tailor their own formal meditation practice by choosing two of the previously trained meditations. The three-minute Breathing Space meditation is also recommended whenever it is needed.

The reminder system: amount 58; contents 3 short videos (the attitudinal foundation of mindfulness: non-judging, and acceptance; Kabat-Zinn, 2015 and “Mindfulness in Everyday Life” by Jon Kabat-Zinn (2017) with Oprah Winfrey at youtube.com) and formal and informal practice reminders for this and other weeks.

Week 8: Your wild and precious life

This week is aimed at helping people to weave mindfulness into daily life to serve as an emergency parachute and to show them some tips on how to develop everyday life mindfulness habits to cultivate it till the end of life.

Pdf file (three pages): recapitulation of the previous week, introduction to the seventh-week content and structure, and description of exercises.

Exercise: tips on how to incorporate mindfulness practice in daily life, it is also suggested to continue with their formal meditation practice choice in week seven. The three-minute Breathing Space meditation is also recommended whenever it is needed.

The reminder system: amount 60; formal and informal practice reminders for this and other weeks.
